# Selective sets of mRNAs localize to extracellular paramural bodies in a rice *glup6* mutant

**DOI:** 10.1093/jxb/ery297

**Published:** 2018-08-09

**Authors:** Yongil Yang, Hong-Li Chou, Andrew J Crofts, Laining Zhang, Li Tian, Haruhiko Washida, Masako Fukuda, Toshihiro Kumamaru, Oliver J Oviedo, Shawn R Starkenburg, Thomas W Okita

**Affiliations:** 1 Institute of Biological Chemistry, Washington State University, Pullman, WA, USA; 2 International Liberal Arts Program, Akita International University, Akita, Japan; 3 Faculty of Agriculture, Kyushu University, Motooka Nishi-ku, Fukuoka, Japan; 4 New Mexico Consortium, Los Alamos, NM, USA

**Keywords:** RNA localization, RNA transport, membrane trafficking, *Oryza sativa*, storage proteins

## Abstract

The transport of rice glutelin storage proteins to the storage vacuoles requires the Rab5 GTPase and its related guanine nucleotide exchange factor (Rab5-GEF). Loss of function of these membrane vesicular trafficking factors results in the initial secretion of storage proteins and later their partial engulfment by the plasma membrane to form an extracellular paramural body (PMB), an aborted endosome complex. Here, we show that in the rice Rab5-GEF mutant *glup6*, glutelin RNAs are specifically mislocalized from their normal location on the cisternal endoplasmic reticulum (ER) to the protein body-ER, and are also apparently translocated to the PMBs. We substantiated the association of mRNAs with this aborted endosome complex by RNA-seq of PMBs purified by flow cytometry. Two PMB-associated groups of RNA were readily resolved: those that were specifically enriched in this aborted complex and those that were highly expressed in the cytoplasm. Examination of the PMB-enriched RNAs indicated that they were not a random sampling of the *glup6* transcriptome but, instead, encompassed only a few functional mRNA classes. Although specific autophagy is also an alternative mechanism, our results support the view that RNA localization may co-opt membrane vesicular trafficking, and that many RNAs that share function or intracellular location are co-transported in developing rice seeds.

## Teaser

Rice mutant lines containing defective membrane trafficking Rab5 and Rab5-GEF mislocalize glutelin RNAs intracellularly, and export specific sets of RNAs that share functions and/or intracellular locations to extracellular paramural bodies.

## Introduction

RNA localization is a ubiquitous and effective strategy employed by micro-organisms, animal, and plant cells in sorting proteins to specific intracellular locations (Martin and Ephrussi, 2009; Chin and Lécuyer, 2017; Eliscovich and Singer, 2017). Such a strategy is employed in developing rice seeds, where mRNAs for the major storage proteins prolamine and glutelin are targeted to specific subdomains of the cortical endoplasmic reticulum (ER) of the bulky seed endosperm (Doroshenk *et al.*, 2011; Tian and Okita, 2014). Prolamine RNAs are localized to the protein body-ER (PB-ER) that delimits the prolamine intracisternal granules, while glutelin RNAs are distributed to the adjoining cisternal-ER (C-ER) (Choi *et al.*, 2000; Hamada *et al.*, 2003*b*). The asymmetric distribution of these storage protein mRNAs facilitates the sorting of the coded protein products (Washida *et al.*, 2004, 2006, 2012). Prolamine polypeptides are retained in the ER lumen while glutelin polypeptides are exported from the ER to the Golgi where they are subsequently transported to the protein storage vacuoles (PSVs).

The transport and localization of the storage protein RNAs to the ER subdomains requires specific cis-sequences (zipcodes) located in the coding sequence and 3′-untranslated region (Hamada *et al.*, 2003*b*; Washida *et al.*, 2009) that are recognized by trans-factors composed of RNA-binding protein complexes (Crofts *et al.*, 2010; Doroshenk *et al.*, 2011, 2012, 2014). Combination of these zipcodes shows that the glutelin RNA transport pathway is dominant over the prolamine RNA transport pathway, which in turn is dominate over a constitutive pathway (Hamada *et al.*, 2003*a*; Washida *et al.*, 2009). Although the three pathways are hierarchal, genetic studies have indicated they are inter-related in that they share common trans-factors (Crofts *et al.*, 2010, Doroshenk *et al.*, 2014, Tian and Okita, 2014).

**Fig. 1.**
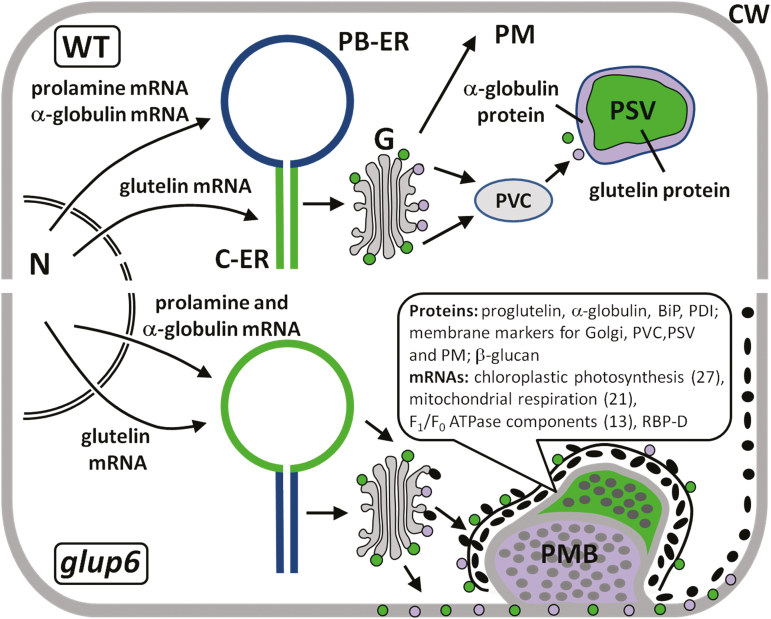
Schematic model showing glutelin mRNA and protein transport in wild-type (WT) and *glup6* mutant rice endosperm. In WT endosperm cells (top), glutelin mRNAs are exported from the nucleus (N) and transported to the cisternal ER (C-ER; shown in green). Following translation, the resulting proglutelins are then exported via the Golgi (G) and pre-vacuolar compartment (PVC) to protein storage vacuoles (PSVs) where they are proteolytically processed to acidic and basic subunits and accumulated (shown in green). In contrast, globulin mRNAs, such as prolamine mRNAs, are targeted to the protein body-ER (PB-ER) and, following export, accumulate at the periphery of the PSVs (shown in purple). Prolamine polypeptides remain in the PB-ER lumen as spherical inclusion granules. In *glup6* (and *glup4*) mutant endosperm cells (bottom), glutelin mRNAs (shown in green) are mislocalized from the C-ER to the PB-ER, whilst prolamine and globulin mRNAs remain correctly localized to PB-ER as in the wild-type. Following export from the ER via the Golgi, both globulin and proglutelin are secreted as small, electron-dense granules to the extracellular space These granules are then partially endocytosed to form novel paramural bodies (PMBs). At subsequent stages, glutelin and globulin are directly transported to the PMBs, together with lumenal chaperones and membrane markers for the Golgi, PVCs, and PSV, as well as β-glucan. *In situ* RT-PCR studies of seed sections and RNA-seq analysis of purified PMBs indicate the presence of RNAs that are either specifically enriched on this sub-compartment or are highly expressed, such as those for glutelin. The coloring of the PMB indicates the typical location of globulin (purple) and glutelin (green) proteins. The major RNA species that are enriched in purified PMBs are indicated. PM, plasma membrane; CW, cell wall.

Although the cis-determinants and several associated RNA-binding proteins have been identified, how the RNAs are transported and targeted to specific locations remains largely undefined. Genetic studies have inferred the involvement of membrane vesicular trafficking in the transport and localization of not only storage proteins to the PSVs but also in the localization of their RNAs (Doroshenk *et al.*, 2010; Fukuda *et al.*, 2016). The GTPase Rab5 and its related guanine nucleotide exchange factor Rab5-GEF are required for membrane trafficking from the plasma membrane (endocytosis) and between the Golgi apparatus and PSVs (Fukuda *et al.*, 2011, 2013). In *glup4* and *glup6* (*glutelin precursor*) mutant lines, proglutelins and α-globulins are secreted to the extracellular space and deposited as small, electron-dense granules during early endosperm development (Fig. 1). These granules are subsequently deposited in the space between the invaginating plasma membrane and the cell wall to form novel paramural bodies (PMBs). Although initially formed by aborted endocytosis of secreted electron-dense granules containing glutelins and globulins, PMBs grow by the apparent direct secretion of endosomal components, including the ER lumenal chaperones BiP and PDI, and membrane-marker proteins for the Golgi, pre-vacuolar compartments (PVCs), and PSVs as well as β-glucan (Fukuda *et al.*, 2011, 2013). Hence, the loss of Rab5 and Rab5-GEF disrupts normal membrane vesicular transport from the ER and Golgi. . In addition to disrupting intracellular trafficking of the protein cargo, RNA localization patterns are also partially altered. Although prolamine and globulin RNAs are correctly targeted to the PB-ER, glutelins RNAs are mislocalized to the PB-ER instead of the C-ER in *glup4* mutants (Doroshenk *et al.*, 2010). In addition, glutelin RNAs are also evident in the PMBs as determined by *in situ* RT-PCR of developing endosperm sections, suggesting that they are transported extracellularly to these novel structures.

Additional evidence for the suggested involvement of membrane vesicular trafficking in RNA localization comes from rice *glup2* lines (Fukuda *et al.*, 2016). These contain a mutation in Golgi transport 1 (GOT1B), a membrane protein that mediates transport between the ER and Golgi. Unlike *glup4*, which mislocalizes glutelin RNAs from the C-ER to the PB-ER, in *glup2* prolamine and globulin RNAs are displaced from the PB-ER to the C-ER.

The mislocalization of storage protein mRNAs in rice lines expressing defective Rab5 and GOT1B suggests that the process of RNA localization is dependent on membrane vesicle trafficking (Doroshenk *et al.*, 2010; Tian and Okita, 2014; Fukuda *et al.*, 2016). Here, we show that, similar to *glup4*, glutelin RNAs are also partially mislocalized to the PB-ER in *glup6*. To directly determine whether PMBs contained RNAs, the aborted endosome complexes were purified by fluorescence-activated particle-sorting by exploiting the selective staining of PMBs with the vital stain fluorescein. Subsequent RNA-seq analysis showed that PMBs possessed two broad classes of mRNAs: those that were specifically enriched with this novel structure and those that were present as minor components compared to their abundant amounts in the cytoplasm. The composition of enriched mRNAs was conspicuously non-random, with the majority resolving into a few functional classes. Our results demonstrate that RNAs that code for functionally related proteins are co-transported to the extracellular PMBs and suggest that this occurs by co-opting membrane vesicular trafficking in developing rice endosperm cells.

## Materials and methods

### Plant growth and selection of mutants

The rice (*Oryza sativa*) *glup6* mutant alleles EM939 and EM1327 were used for RNAseq analysis of purified PMBs and microscopy studies, respectively (Kumamaru *et al.*, 1988; Fukuda *et al.*, 2013). EM939 plants were grown at Washington State University in a controlled environment chamber with a day/night settings of 26/22 °C and 11/13 h at a light intensity of 560–700 µmol m^–2^ s^–1^. EM1327 plants were grown in the field at Kyushu University.

### Immunofluorescence and transmission electron microscopy

Developing seeds of *glup6* line EM1327 were fixed, sectioned, and analysed by immunofluorescence or by electron microscopy as described previously by Fukuda *et al.* (2013).

### RNA preparation, sequencing, and analysis

Total RNA was extracted using a protocol modified from Wang and Vodkin (1994). Three biological replicates (six seeds each from three individual plants) were prepared from developing seeds 14 d after flowering (DAF) for the wild-type Taichung 65 (TC65) and the *glup6* mutant. The seeds of each sample were dehulled, pooled, and ground in liquid nitrogen. Each powdered sample was suspended in 80 mM Tris, 16 mM EDTA, 160 mM NaCl, 4% SDS, and 16 mM DTT, and then extracted with an equal volume of buffer-saturated phenol. The mixture was centrifuged, the upper aqueous phase was extracted with chloroform, and the RNA was precipitated overnight at 4 °C overnight in the presence of 2 M LiCl, followed by a second precipitation in ethanol. After centrifugation, the resulting RNA pellets were washed in 70% ethanol and then dissolved in diethyl pyrocarbonate (DEPC)-treated water.

The concentration and quality of each RNA sample were determined using a ThermoFisher Qubit RNA HS Kit and an Agilent Bioanalyzer 6000 Pico Chip, respectively. Polyadenylated (poly-A) RNA was enriched from the tRNA using a NEBNext Poly(A) mRNA Magnetic Isolation Module. The concentration of the poly-A-enriched RNA was obtained using the Qubit RNA HS Assay and an aliquot of 50 ng was fragmented and converted to cDNA. Adapter sequences and indexes were then added to the ends of the fragments to generate libraries for sequencing using an Illumina NextSeq 500. The resulting sequencing library for each sample was denatured to a final concentration of 1.5 pM before being loaded onto a NextSeq Reagent Cartridge for a paired-end 151-bp sequencing run.

The recovered forward and reverse 2 × 151-bp Nextseq reads were concatenated into a single file and mapped as single-end reads for analysis (Yang *et al.*, 2018). All sequence reads were trimmed for quality using the FastQ Quality Control Software (Chien-Chi and Chain, 2014). All reads with a minimum sliding window *q*-score of 15, and a minimum trimmed read length of >29 bp were kept for mapping and analysis. Quality-filtered reads were mapped using Bowtie2 (http://bowtie-bio.sourceforge.net/bowtie2; accessed 22 August 2018) with default settings, according to the manual. These included end-to-end alignment, default alignment scores (+/–0.6 × read length), and the default mode of searching for multiple alignments and then reporting the best one, or choosing randomly among equally good alignments. Abundance estimation (RPKM, fragments per kilobase of exon per million mapped reads) was calculated using RSEM (Li and Dewey, 2011). Differential expression of transcripts was determined using EdgeR (https://bioconductor.org/packages/release/bioc/html/edgeR.html; accessed 22 August 2018). Transcripts that were differentially expressed >2-fold with a *P*-value <0.001 were considered statistically significant.

### Fluorescein staining and purification of PMBs by flow cytometric analysis

One hundred developing seeds (mainly at 10–12 DAF) were freshly collected and dehulled. In order to prepare ‘seed peels’, the pericarp was removed using tweezers and then the seeds were sliced longitudinally using a thin razor blade, leaving 1 mm of tissue uncut (so that the seeds resembled a book with the uncut portion being the spine; see Supplementary Fig. S1 at *JXB* online). The two halves of the seed were then opened up and the soft, bulky endosperm tissue removed to leave the tegmentum, the aleurone cell layer, and 3–5 layers of subaleurone intact. These seed peels were then stained overnight with 1:1000 diluted fluorescein-labeled anti-rabbit secondary antibody at 4 °C. The stained seed peels were washed three times with cold 1× PBS (137 mM NaCl, 10 mM phosphate, 2.7 mM KCl, pH 7.4) for 30 min to remove excess staining. Stained dilated structures (the PMBs), which are located in the subaleurone layers, were released from membranes by sonication: two stained aleurone peels were submerged in 500 µl of 1× PBS in a 2-ml microfuge tube and sonicated three times with 5 W for 20 s. The supernatant, now containing the released PMBs, was pooled and then subjected to sorting by flow cytometry.

The Becton Dickinson (San Jose, CA) FACS Vantage with Diva software operating system was used to isolate fluorescent PMBs. A selective electronic gate with side and forward light scattering was used to select for particle size and a second gate was used to select for fluorescence. A purified PMB fraction was readily collected by comparing the particle-size fluorescence spectra generated by resolved preparations from *glup6* and wild-type aleurone/subaleurone peels (see Results). A 10-ml fraction of size- and fluorescence-selected PMB particles was concentrated to 1 ml final volume using a Centricon 100 kDa centrifugal filter. Overall, three biological replicates were prepared over a 10-d period.

### RNA purification from PMBs and RNA-seq

Half of the purified PMB fraction was incubated with 2 units of RNase free DNase I (Promega) for 30 min at 37 ºC to remove potential contamination by genomic DNA. RNA was isolated from purified PMBs by mixing with 500 µl of TRIzol reagent (Invitrogen) followed by centrifugation at 13 400 *g* for 10 min. The supernatant was extracted with 100 µl of chloroform and centrifuged for 10 min. To improve RNA quality, 400 µl of the upper aqueous phase was mixed with 400 µl of 70% EtOH and then further purified using a RNeasy Mini Purification Kit (Qiagen). The integrity and purity of total RNA were assessed using an Agilent Bioanalyzer and OD260/280 with a Nanodrop spectrophotometer. Using 100 ng of total RNA, 1–2 μg of cDNA was generated using a Clontech Smart cDNA Synthesis Kit (Clontech Laboratories, Inc.). cDNA was fragmented using a sonicator (Covaris, Inc.), profiled using an Agilent Bioanalyzer, and subjected to Illumina library preparation using NEBNext reagents (New England Biolabs).

For control transcripts, total RNA was extracted from seed peels isolated from developing wild-type TC65 seeds (10–15 DAF) using the RNA extraction method described above. In detail, five seed peels were ground to a fine powder under liquid nitrogen and then transferred to a tube containing 500 ul Extraction Buffer (80 mM Tris, 16 mM EDTA, 160 mM NaCl, 4% SDS, and 16 mM DTT). An equal volume of buffer-saturated phenol was added, mixed well, and centrifuged at 13 400 *g* at 4 °C for 15 min. The upper phase was transferred to a new tube and an equal volume of chloroform was added before centrifuging again at 4 °C for 15 min. The upper aqueous phase was then collected and total RNA precipitated by incubation with an equal volume of 4 M LiCl at 4 °C overnight. The RNA was pelleted by centrifugation at 4 °C for 30 min, resuspended in DEPC-treated water, and then precipitated in the presence of 0.3 M NaOAC (pH 5.2) and 2.5 volumes of 95% ethanol. The mixture was frozen in liquid nitrogen for 1 min and centrifuged at 4 °C for 30 min. The RNA pellet was washed in 70% ethanol after discarding the supernatant and, after air-drying, was then dissolved in 10 μl DEPC-treated water. A 10-ng sample of total RNA was subjected to double-strand cDNA amplification. The cDNA library was loaded on a HiSeq 2000 instrument and paired-end sequencing with 100 base reads was performed. The processing of fluorescent images into sequences, base-calling, and quality value calculations were performed using the Illumina data processing pipeline (version 1.8). Read sequences were aligned and mapped to the MSU v.7.0 cDNA reference (http://rice.plantbiology.msu.edu/; accessed 22 August 2018) using CLC Genomics Workbench (v.5.05; Qiagen). To profile transcript expression levels for each mapped gene, RPKM was calculated for the exons of the transcripts in the respective MSU v.7.0 annotation. We only selected valuable transcript species for which cDNA species possessed an RPKM value ≥1 in both wild-type and sorted samples.

### *In situ* RT-PCR

The procedure used for cryosectioning, fixation, and washing of sections of 10–15-d-old developing rice seed prior to *in situ* RT-PCR was as described previously by Choi *et al.* (2000). *In situ* RT-PCR was performed in the presence of 200 µM each of dATP, dCTP, dGTP, 190 µM dTTP, and 20 µM Alexa Fluor 488–dUTP, with basic PCR ingredients including 3 mM MgCl_2_, 0.5 mM MnSO_4_, 5 mM dithiothreitol, 2.5 U Tth DNA polymerase (Epicentre, Madison, WI, USA), and 1× PCR Enhancer. The primers used for cDNA synthesis are listed in Supplementary Table S9. These gene-specific primers were used at a final concentration of 0.5 µM. Prepared sections were immersed in PCR reaction mixture and reverse transcription was allowed to proceed at room temperature for 30 min followed by 60 °C for 30 min. Sections were then subjected to 12 cycles of 94 °C for 1 min; 54 °C, 55 °C, or 56 °C for 1 min; 72 °C for 1.5 min; followed by 72 °C for 5 min. Following RT-PCR, sections were washed twice for 2 h each time in 2× SSC (0.3 M NaCl, 30 mM sodium citrate) and twice for 10 min each in 0.2× SSC. PB-ER was labeled by adding 0.1 µM Rhodamine B hexyl ester with brief washing with PBS (137 mM NaCl, 10 mM Na^+^ phosphate, 2.7 mM KCl, pH 7.4) to remove excess staining. Samples were observed using a Zeiss 510 series laser-scanning confocal microscope (Jena, Germany). Image processing was performed using Adobe Photoshop, NIH Image (https://imagej.nih.gov/nih-image/; accessed 22 August 2018), or Microsoft PowerPoint.

## Results

### Dilated multivesicular body-like structures (PMBs) in *glup6* seeds

**Fig. 2.**
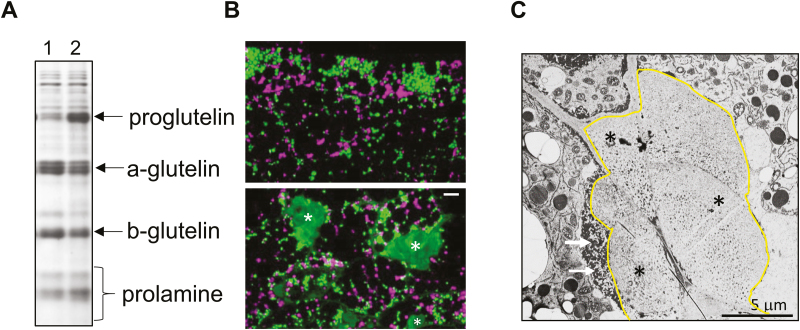
Properties of the rice *glup6* mutant lines. (A) SDS-PAGE showing the over-accumulation of proglutelin and reduction in glutelin acidic and basic subunits in the *glup6* rice mutant (lane 2) as compared to the wild-type (lane 1). (B) Distribution of prolamine (purple) and glutelin polypeptides (green) in the wild-type (upper panel) and in *glup6* (lower panel). In the wild-type, prolamine polypeptides are deposited as intracisternal granules within the ER while glutelins are transported to the protein storage vacuoles. In the wild-type the bulk of storage protein synthesis and accumulations clearly occurs in the outer endosperm (subaleurone) layers. In *glup6*, glutelins are initially secreted to the cell periphery where they are partially endocytosed to form paramural bodies (PMBs, labeled with asterisks). As the seed develops, glutelins and other endosomal proteins are directly transported and secreted to these novel structures. (C) Transmission electron micrograph of *glup6* endosperm depicting a large PMB (outlined in yellow) formed at the junction of three endosperm cells. The arrows show the large number of electron-dense granules bordering the PMB.

Like their counterpart Rab5-defective *glup4* plant line, developing *glup6* seeds exhibited the same defects in storage-protein accumulation and endosomal trafficking. This mutant line, defective in the Rab5-dependent GEF, over-accumulated the 57-kD proglutelin precursor due to a defect in its transport to the protein storage vacuoles (PSVs), where it would normally be processed to acidic and basic subunits (Fig. 2A). Instead, a significant amount of the synthesized proglutelin was transported to the plasma membrane where it was secreted and then subsequently partially taken up by an aborted endocytosis process to form paramural bodies (PMBs) (Fig. 2B, C). Subsequent growth of the PMBs apparently occurs by direct extracellular translocation and deposition of proglutelin and α-globulin storage proteins to them (Fukuda *et al.*, 2011, 2013). Moreover, the ER markers BiP and PDI (PDIL1), the Golgi marker β-1,4-galactosyltransferase, the PVC markers VSR3 and PV72, and the PSV marker α-TIP, are also prominently detected in the PMBs (Fukuda *et al.*, 2011). The location in the PMBs of these endosomal marker proteins, many of them membrane proteins, indicate considerable displacement of endosomal proteins to the PMBs due to the disruption in membrane trafficking from the ER to the PSVs.

**Fig. 3.**
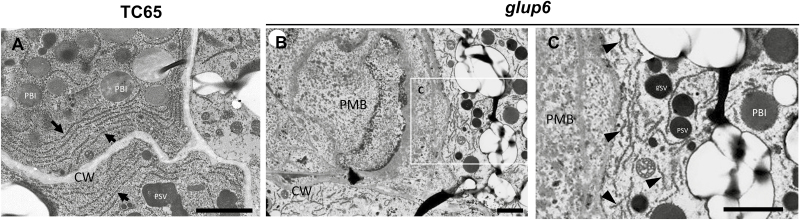
Transmission electron micrographs depicting the ER in wild-type and *glup6* endosperm. (A) Wild-type TC65 and (B, C) *glup6*. (C) Shows an enlarged image of the area indicated in (B). The arrows in (A) indicate the layers of cisternal-ER membrane in the wild-type. The arrowheads in (C) indicate the dispersed ER in *glup6*. Scale bars are 2 μm.

The loss of Rab5 and GEF dramatically alters the morphology of the ER. Under light microscopy, the polygonal tubular-cisternal network of ER membranes with associated prolamine-containing protein bodies, a prominent feature in the wild-type, is never observed in *glup4* (see supplementary fig. S7 of Fukuda *et al.*, 2011) or *glup6* (unpublished results). At the TEM level, the ER is organized as successive layers of membrane at the cortex of the cell in wild-type endosperm cells. In *glup6*, the ER membranes were distributed in a more disordered manner near the PMBs and were far less abundant (Fig. 3) than observed in the wild-type.

**Fig. 4.**
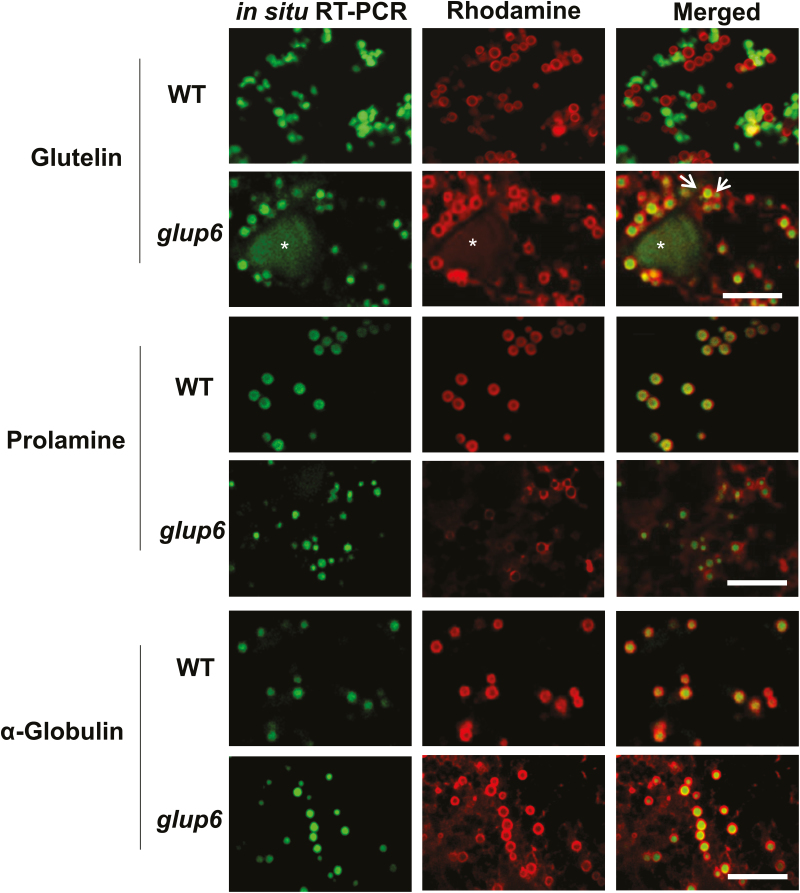
Localization of glutelin, prolamine, and α-globulin RNAs in wild-type (WT, TC65) and *glup6* mutant lines. In *glup6*, glutelin (LOC_Os01g55690) RNAs are mislocalized to the protein body-ER (PB-ER; ringlet structures labeled red by Rhodamine) and paramural bodies (PMBs; indicated with asterisks), while prolamine (LOC_ Os07g10570) and α-globulin (LOC_Os05g41790) RNAs are distributed to their normal locations on the PB-ER. It should be noted that in many instances RNAs localized to PB-ER, such as α-globulin RNAs (bottom panels), are observed as deposits instead of being distributed to the periphery of the prolamine protein body This is an artefact probably caused by the high temperature used in the *in situ* RT-PCR procedure, which apparently disrupts the integrity of the ER membrane and causes it to condense as a particle The intense Rhodamine staining of the PB-ER compared to the cisternal-ER is also contributed by its binding to the surface of the hydrophobic prolamine inclusion granule. Scale bars are 5 μm.

In addition to the aberrant endosomal trafficking of secretory proteins, the transport and localization of storage-protein RNAs was also disrupted in *glup6* (Fig. 4), as seen previously in *glup4* (Doroshenk *et al.*, 2010). Unlike in wild-type cells where glutelin RNAs were localized to the C-ER, they were found to be mislocalized to the PB-ER and on the PMBs in *glup6*. In contrast, prolamine and α-globulin RNAs were targeted to their usual location on the PB-ER. Hence, transport and localization of glutelin RNAs was dependent on the membrane trafficking factors Rab5 and Rab5-GEF.

### Loss of Rab6-GEF alters the transcriptome in the *glup6* mutant

To assess the changes in gene expression at the RNA level, the transcriptomes of mid-stage developing seeds from the wild-type and *glup6* were obtained by shotgun Illumina sequencing of polyadenylated RNA transcripts. After processing, the trimmed reads were aligned against the MSU v.7.0 pseudomolecule cDNA reference and normalized to RPKM for assessment of differential transcript abundance. While the transcriptomes of the three biological replicates of the wild-type or *glup6* were highly correlative (*R*^2^=0.96), they were clearly distinct from each other (*R*^2^=0.49). In general, *glup6* seeds exhibited higher transcriptional activity than wild-type seeds. Considering RNAs having a RPKM≥1.0, seeds of *glup6* had on average 11784 RNAs at this abundance while the wild-type had a significantly lower number at 6676 (Supplementary Table S1). This increase in transcriptional activity in *glup6* was due to the activation of specific genes. Using an RPKM value of ≥1.0 as a baseline, 3955 transcripts were preferentially expressed 2-fold or higher in *glup6* compared to the wild-type (Table 1, Supplementary Table S2A). From this total, 270 were expressed only in *glup6* and, therefore, were specifically transcriptionally activated in this mutant line (Table 1, Supplementary Table S2B). The largest class of up-regulated RNAs coded for expressed/hypothetical proteins (>1100), followed by enzymes (621), membrane proteins (322), signal transduction-related proteins (252), DNA-/RNA-binding proteins (217), ABA/stress-related proteins (209), and retrotransposons/transposons (195). Consistent with the major gene activation and elevated transcriptional activity, more than 170 transcription factors (TFs) were up-regulated with eight being specifically activated only in *glup6* and silenced in the wild-type (Table 1). Except for two, none of the TFs have been defined molecularly. The ‘no apical meristem’ TF controls boundary formation at meristems and lateral organ separation in *Medicago truncatula* and Petunia (Souer *et al.*, 1996; Cheng *et al.*, 2012). The ethylene-responsive transcriptional factors of Arabidopsis bind to the GCC-box of stress-related genes (Fujimoto *et al.*, 2000). Eight other ‘no apical meristem proteins’ and five additional ethylene-responsive TFs were activated by at least 2-fold or more in *glup6*. RNAs coding for protein turnover and protein modification as well as those associated with energy transfer (respiration and photosynthesis) were also significantly up-regulated (Table 1).

**Table 1.** Up- and down-regulated transcripts in *glup6* compared to the wild-type (WT)

Class	RPKM_*glup6*_≥1.0			RPKM_WT_≥1.0		
	RPKM_WT_=0	*glup6*/WT≥5.0	5.0≥*glup6*/WT≥2.0	RPKM_*glup6*_=0	WT/*glup6*≥5.0	5.0≥WT/*glup6* ≥2.0
ABA and stress	24	68	117	1	1	15
Antimicrobial peptides	1	39	13	1	1	4
Cell wall-related proteins	1	8	13	0	0	5
Cytoskeleton-related	0	2	24	0	0	0
DNA-/RNA-binding proteins	2	32	183	0	6	14
Energy: Respiration and photosynthesis	8	6	36	0	1	2
Enzymes	10	142	469	2	5	33
Expressed/hypothetical/unknown proteins	136	231	745	30	26	114
Membrane dynamics and transporters	6	75	241	1	5	33
Protein turnover – modification	6	15	60	1	1	5
Ribosomal proteins	3	5	18	1	1	8
Signal transduction	6	54	192	0	5	23
Storage proteins	1	4	20	0	0	3
Transcription factors	8	51	112	1	1	16
Retrotransposons/transposons	37	36	122	8	20	46
Miscellaneous	21	113	439	3	5	25
**Subtotal transcripts count**	**270**	**881**	**2804**	**49**	**78**	**346**
**TOTAL**	**3955**			**473**		

The number of RNAs in each class are presented at a RPKM of ≥1.0 and at a RPKM ratio of *glup6*/WT ≥2.0 for up-regulated RNAs, and RPKM ratio WT/*glup6* ≥20 for down-regulated RNAs.

Substantially fewer genes (473 in total) were down-regulated in *glup6* compared to the wild-type (Table 1, Supplementary Table S3). In general, the same RNA classes were affected, with expressed/hypothetical proteins being the largest class (170) that was transcriptionally-suppressed in *glup6*, followed by retrotransposons/transposons (74). The expression of a single TF, NAC domain-containing protein, was silenced in *glup6*.

### Preparation and isolation of fluorescently labeled PMBs

**Fig. 5.**
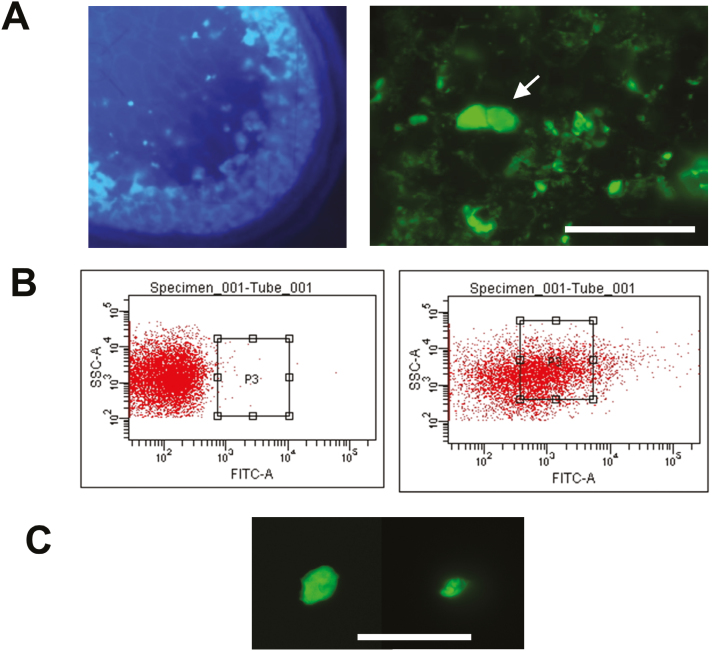
Properties of paramural bodies (PMBs) and their purification by flow cytometry. (A) Calcufluor staining of a *glup6* endosperm section. Callose-containing PMBs are distributed mainly to the outer subaleurone cell layers of the developing endosperm (left panel). The right panel depicts the labeling of PMBs (two adjacent large PMBs are indicated by an arrow) when aleurone/subaleurone peels were incubated with fluorescein-conjugated anti-rabbit antibody. (B) Flow cytometry fractionation of wild-type (left panel) and *glup6* (right panel) extracts isolated from aleurone/subaleurone peels. PMBs were isolated using a combination of size and fluorescence signal strength. (C) Fluorescein-labeled PMBs distributed in the boxes indicated in the right panel in (B) were collected and observed by microscopy. Scale bars are 10 μm.

The localization of glutelin RNAs, but not prolamine or α-globulin RNAs, on the PMBs (Fig. 4) suggested that selected RNAs were transported extracellularly to the PMBs. To eliminate the possibility that this apparent localization of glutelin RNAs was an artifact caused by non-specific labeling by the fluorescently labeled nucleotide during the *in situ* RT-PCR reaction of endosperm sections, we set out to purify the PMBs. Having observed abnormally high background binding of fluorescein-labeled secondary antibodies to the PMBs, the ‘sticky’ nature of these structures was exploited to allow their rapid and efficient purification by fluorescence-activated cell sorting (FACS) and, in turn, to permit the identification and quantification of PMB-associated mRNA species. The starting materials chosen for this procedure were ‘aleurone/subaleurone peels’ isolated from mid-developing seeds of both the wild-type (TC65) and *glup6* (Fig. 5A, Supplementary Fig. S1). These seed peels included the tegmentum, a single aleurone cell layer, and 3–5 subaleurone cell layers of the bulky endosperm. The subaleurone layers were highly enriched in PMBs (Figs 2B, 5A) due to elevated storage-protein expression in these outer endosperm cell layers. A four-step procedure was thus established in which seed peels were incubated with fluorescein-labeled goat anti-rabbit antibody, washed to reduce background signal, sonicated to release the labeled PMBs, and then subjected to fluorescence-activated particle-sorting (FAPS) to enrich for particles by size and fluorescence (Fig. 5B). In contrast to material derived from *glup6* in which PMBs could be readily stained and sorted by fluorescence intensity and particle size, very little fluorescence was observed or collected by FAPS using wild-type material, clearly demonstrating the validity of this approach (Fig. 5B). The purification of PMBs by FAPS was verified by fluorescence microscopy (Fig. 5C). Using the FAPS-purified PMBs as staring material, ~500 ng of total RNA was recovered from each of two preparations. A third sample failed to provide sufficient RNA for further analysis.

### RNAs associated with *glup6* PMBs are distinct from the wild-type transcriptome

RNA samples (~10 ng each) from two purified PMB samples were reverse-transcribed and the resulting cDNA was amplified using Ovation RNA-seq System V2 to yield 5.9 ± 1.6 μg. We obtained a total of 29 million reads (19456436 and 9624850) for *glup6*-PMB by Illumina sequencing. After processing, the trimmed reads were aligned against the MSU v.7.0 pseudomolecule cDNA reference and normalized to RPKM for assessment of differential transcript abundance between the wild-type and *glup6*. While the biological replicates of the PMB cDNA libraries exhibited moderate correlation (*R*^2^=0.68), the two PMB cDNA libraries showed poor correlation (*R*^2^=0.15) of gene expression with the *glup6* transcriptome, indicating that the PMB libraries were considerably distinct from *glup6*. This diversity was also reflected in the number of gene sequences with RPKM values ≥1.0. While the *glup6* transcriptome had 11784 gene sequences with a RPKM ≥1.0, a number close to the reported rice endosperm transcriptome (Gao *et al.*, 2013), the PMBs only contained 2316 gene sequences having RPKM values ≥1.0, indicating that the PMB cDNA library was highly enriched for a subset of mRNAs (Supplementary Table S1). One conspicuous anomaly was the substantial abundance of two RNAs coded by LOC_Os09g00999 and LOC_Os09g01000, which had RPKM values >88000. Although these RNAs were annotated as expressed proteins in the MSU v.7.0database, both contained abundant repetitive sequences with significant alignment with 18S and 26S rRNA sequences. These alignments were evident over their entire sequences (Supplementary Table S4). The predominance of 18S and 26S rRNAs suggested that ribosomes comprised a major proportion of the RNAs associated with the purified PMBs.

### Specific RNA classes are enriched in the purified PMB fraction

To identify mRNA sequences that were enriched in the PMBs, we selected PMB-localized RNA sequences present with an RPKM value ≥1.0 and enriched over the total *glup6* transcriptome (PMB/*glup6*) by at least 5-fold or greater. Based on these criteria, the differentially expressed PMB transcriptome contained 1245 RNAs, the majority consisting of expressed proteins (446), hypothetical proteins (149), retrotransposons (331), and transposons (149) (Table 2, Supplementary Table S5). The remaining 170 RNA sequences were clearly non-random and coded for proteins involved in specific cellular functions (Tables 2, 3). The two largest classes were energy-related; one for chloroplast photosynthesis (27) and the other for mitochondrial respiration (21). The photosynthesis class contained subunit sequences for both photosystems (PS) I and II. RNAs coding for the PSI apparatus were those for the iron-sulfur center (3), P700 chlorophyll *a* aproprotein (4), and two PSI assembly proteins, ycf3 and ycf4 (Supplementary Table S6). Additional RNAs coding for PSII proteins included cytochrome b559 (2 genes), D1 and D2 proteins, P680 chlorophyll *a* apoprotein (4 genes), and reaction the center proteins psbC and psbK. Four PMB-enriched RNAs coded for proteins that transfer electrons from PSII to PSI. These included those for the cytochrome b6-f complex including cytochrome b6 (3 genes) and the cytochrome b6-f complex subunit 4, and for plastocyanin. Lastly, RNAs for NADPH-dependent oxidoreductase (7) and the CO_2_-fixing enzyme Rubisco large subunit (2) were also enriched on the PMBs.

**Table 2.** RNA classes enriched in paramural bodies (PMBs)

Class	No.
ABA and stress	0
Antimicrobial peptides	8
Cell wall-related	10
Cytoskeleton	1
DNA binding	17
Energy: Photosynthesis	33
Energy: Respiration	17
Enzymes	5
Expressed proteins	446
Hypothetical and unknown proteins	149
Membrane (mainly transporting activity)	6
Protein turnover and modification	4
Retrotransposons	331
Ribosomal proteins: cytoplasm	3
Ribosomal proteins: mitochondria	4
Ribosomal proteins: plastid	9
RNA binding proteins	8
Signal transduction	3
Starch biosynthesis	0
Storage proteins	0
Transcription factors	5
Transposons	149
Miscellaneous	38
**Total**	**1246**

Number of RNAs in each class present in PMBs at RPKM ≥1.0 and enriched PMB/*glup6* ≥5.0.

**Table 3.** RNAs associated with paramural bodies (PMBs) of RPKMs ≥5.0

Class		PMB/*glup6*		
	Total	≥ 1.0	0.2-0.99	<0.20
ABA and stress	6	0	0	6
Antimicrobial peptides	3	1	1	1
Cell wall-related	0	0	0	0
Cytoskeleton	4	1	1	2
DNA-binding	7	4	1	2
Expressed proteins	75	63	5	7
Energy: respiration	18	18	0	0
Energy: photosynthesis	29	29	0	0
Enzymes	4	1	2	1
Hypothetical and unknown proteins	29	29	0	0
Membrane (mainly transporting activity)	7	1	0	6
Protein turnover and enzymes	6	4	0	2
Retrotransposons	24	22	2	0
Ribosomal proteins: cytoplasm	4	2	2	0
Ribosomal proteins: mitochondria	5	5	0	0
Ribosomal proteins: plastid	15	11	0	4
RNA binding proteins	8	4	0	4
Signal transduction	4	0	0	4
Storage proteins	28	1	2	25
Transcription factors	3	0	1	2
Transposons	19	19	0	0
Miscellaneous	15	9	2	4
**Total**	**313**	**220**	**21**	**72**

 Of the 17 RNAs in the energy-respiration class, 13 coded for various subunits of the F_1_ and F_0_ complexes of ATPase synthase. These included the A, B, and C subunits of the F_0_ membrane proton channel and two (α and β) of the five subunits of the F1 catalytic core. Two RNAs coded for proteins in cytochrome C oxidase formation and two coded for subunits of the NADH-ubiquinone oxidoreductase.

Transcripts in the DNA-binding class (17) coded for proteins involved in RNA transcription and DNA replication and repair, although many RNAs in this class coded for DNA-binding proteins of unknown functions. Two RNA-binding classes were evident: one coded for ribosomal proteins associated with cytoplasmic (3), mitochondrial (4), and plastid (9) ribosomes, and the second included RNA-binding proteins. One notable RNA-binding protein RBP-D (LOC_Os06g37000) recognizes the prolamine zipcode and therefore probably participates in the transport and localization of prolamine RNAs to the PB-ER. The expression of RBP-D was only slightly activated (1.4-fold) in *glup6* compared to the wild-type but it was enriched more than 33-fold in the PMBs (Supplementary Table S6). A second RNA-recognition motif (RRM)-containing RNA-binding protein (LOC_Os03g04780) that was normally transcriptionally silent in the wild-type was also prevalent in PMBs.

Two other classes enriched on the PMBs were cell wall-related and membrane-related proteins. The cell wall-related class consisted of eight glycine-rich cell wall structural proteins, cellulose synthase-like protein, and lignin-degrading laccase-9 precursor. Six of the RNAs for glycine-rich cell wall structural proteins were activated in *glup6* and specifically found only in the PMBs. The membrane-related class consisted mainly of transporters for small molecules (e.g. aquaporin) or polypeptides (tat-like protein). The gene activation of cell wall- and membrane-related proteins was consistent with the significant changes at the cell surface due to the formation of PMBs.

Other RNAs coded for antimicrobial peptides (8), enzymes (6), cytoskeleton (1), protein turnover-modification (4), signal transduction (3), and a large class of miscellaneous RNAs (37). Many of the RNAs in this latter class coded for proteins with defined peptide domains (e.g. F-box domains, glycine-rich). Sixteen were transcripts for latency-associated nuclear antigens.

### Sets of RNAs present on PMBs with RPKM≥5.0

Although glutelin RNAs were conspicuously localized on the PMBs, as observed by *in situ* RT-PCR, they were not enriched relative to their *glup6* levels. Examination of the RNA-seq data, however, indicated that eight glutelin RNAs were among the first 40 most-abundant RNAs on the PMBs (Supplementary Table S1). Analysis of the RPKM-based PMB/wild-type ratio showed that glutelins were distributed to the PMBs mainly due to their very high expression during seed development. For example, glutelin RNA (LOC_Os01g55690) had a RPKM value of ~110 in the PMB fraction but this constituted less than 0.5% of the total expressed in the *glup6* transcriptome.

As PMBs appeared to contain RNAs that were abundantly expressed, we cataloged RNAs that were present on the PMBs with a RPKMvalue ≥5.0 (Supplementary Table S7). At this relative abundance, the number of RNAs associated with the PMBs was substantially lower than the PMB-enriched RNAs listed in Table 2. This reduction was mainly due to the significantly lower number of RNAs that coded for expressed and hypothetical proteins, retrotransposons, and transposons having RPKM values ≥5.0 in the PMB fraction. For example, while 331 retrotransposon RNAs were found to be enriched 5-fold or more in PMBs compared to the total *glup6* transcriptome (Table 2), only 22 had RPKMs ≥5.0 in the PMB fraction. The bulk of the RNAs coding for expressed and hypothetical proteins, retrotransposons, and transposons (Supplementary Table S5), however, were also found to be enriched (PMB/*glup6*≥5.0) in the PMBs (Table 2, Supplementary Table S6).

The other major change clearly evident was the presence of storage-protein RNAs, consisting of 11 glutelin RNAs, one albumin, and one cupin. In addition, substantial quantities of 14 prolamine RNAs and one α-globulin were also present. RPKM values ranged from a high of >202 for one of the glutelin RNAs (LOC_Os02g15169) to a low of ~5 for another glutelin RNA (LOC_Os02g15070). Other significant differences are observed for ABA stress- and cell wall-related RNAs. Six prominent RNAs that coded for heat-shock proteins and other stress-related proteins had prominent RPKM values in the PMBs, although they encompassed only 1–6% of the total transcriptome levels in *glup6*. No cell wall-related RNAs were present at a RPKM value ≥5.0.

Other than the changes observed for the aforementioned classes of RNAs, many of the other RNA classes were relatively unaffected by the two criteria used in Tables 2 and 3. This was most conspicuous for the energy-related respiration and photosynthesis classes. Nearly all of the RNAs in these two classes were abundant and enriched in the PMBs.

### Mislocalization of novel mRNA species to the PB-ER and accumulation on dilated structures

**Fig. 6.**
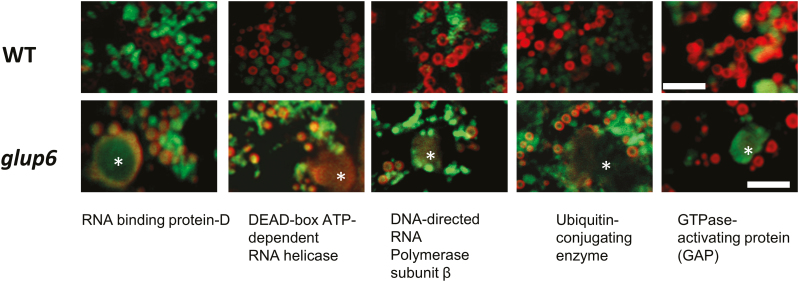
Mislocalization of mRNAs encoding RBP-D, DEAD-box ATP-dependent RNA helicase, RNA polymerase subunit b, ubiquitin conjugating enzyme, and GTPase-activating protein in the *glup6* mutant compared with the wild-type (WT). All panels represent merged images of endosperm sections where Rhodamine staining (red) has been superimposed on the fluorescence staining patterns generated by *in situ* RT-PCR depicting the distribution of each individual RNA (green). With the exception of GTPase-activating protein, the mRNAs are mislocalized to the protein body-ER (PB-ER) and to the paramural bodies (PMB; indicated with asterisks) in the *glup6* endosperm. The transcript for GTPase activating protein is found on the cisternal-ER and PMBs, and is not observed on the PB-ER in *glup6*. See Supplementary Fig. S2 for individual Rhodamine staining and RNA localization distribution patterns generated by *in situ* RT-PCR. Scale bars are 5 µm.

To validate the mislocalization of RNAs to the PMBs, selected RNAs were subjected to analysis by *in situ* RT-PCR. These RNAs varied in abundance on the PMBs from RPKM values >150 for RBP-D to <13 for DNA-directed RNA polymerase (Supplementary Table S1). As shown in Fig. 6 and Supplementary Fig. S2, RNAs for RBP-D, DEAD-box RNA helicase, DNA-dependent RNA polymerase subunit, ubiquitin-conjugating enzyme, and GTPase-activating protein were normally localized to the C-ER in the wild-type. In *glup6*, other than the RNA for GTPase-activating protein, the localization patterns were identical to those seen for glutelin RNAs in *glup6*; i.e. in addition to the C-ER, they were partially mislocalized to the PB-ER and also to the PMBs. Interestingly, RNAs coding for the GTPase-activating protein were observed mainly with the PMBs, with very little located intracellularly. Unlike the other RNAs examined, the one coding for GTPase-activating protein was not associated with the PB-ER.

## Discussion

The loss-of-function of Rab5 and Rab5-GEF activities disrupts normal membrane trafficking during the mid-stage of rice seed development (Fukuda *et al.*, 2013). Initially, cargo vesicles containing storage proteins are diverted from the storage vacuole to the plasma membrane, whereupon the storage proteins are secreted into the intercellular space. These proteins are subsequently partially engulfed by an aborted endosomal process to form a prominent extracellular PMB (Fig. 1). PMBs also contain proteins normally located in the ER, Golgi, PVC, and PSVs. The secretion of these proteins, many of them membrane proteins, to the PMB indicates that normal membrane vesicular trafficking from the ER to the PSVs is blocked. The presence of numerous membranous electron-dense granules bordering the cytoplasmic face of the PMBs (Fig. 2C) and the absence of extracellular glutelin granules in the cell wall space suggest the direct transport of membrane vesicles to the PMB surface, and subsequent accumulation of their cargo within the PMB.

Under normal conditions there is a balance between outward and inward trafficking of membrane vesicles between the ER and PSVs. An indirect consequence of outward membrane trafficking to the PMBs in *glup6* (as well as *glup4*) is the reduction in intracellular membranes. This was readily apparent for the ER, which under normal conditions was arranged in multiple layers, and ran parallel to the plasma membrane (Fig. 3A). In *glup6*, the distribution of the ER was random in relation to the plasma membrane and the overall content was lower than that seen in the wild-type (Fig. 3B, C). The reduction in the ER was probably the result of outward transport not being balanced by inward membrane trafficking.

Our results demonstrated that the loss-of-function of Rab5-GEF in *glup6* also disrupted the normal intracellular distribution of selective mRNAs in developing rice endosperm. While prolamine and globulin RNAs were correctly targeted to the PB-ER, glutelin mRNAs were mislocalized to this ER subdomain (Fig. 4). The normal localization of prolamine and globulin RNAs on the PB-ER indicated that the consequences of mutations in Rab5 and Rab5-GEF were selective and were not due to a total disruption of the integrity of the cortical ER.

Examination of storage-protein RNA distribution patterns indicated that glutelin RNAs were also observed associated with PMBs. Direct evidence for the presence of PMB-associated RNAs was obtained by RNA-seq analysis of PMBs purified by fluorescence-activated particle sorting. Two classes of RNAs were clearly present in the PMBs: one consisted of RNAs that were specifically enriched within the PMBs (Supplementary Table S6) the second consisted of highly expressed RNAs where only a small proportion were associated with the PMBs (Table 3). Highly expressed RNAs such as those for glutelin were, in the most part, retained by the cell and only a small portion were extracellularly transported to the PMBs. These observations indicated that Rab5 and Rab5-GEF were not only required for RNA transport and localization, but that they may also have distinct roles depending on the RNA species. While glutelin RNAs were mislocalized intracellularly, RNAs enriched on the PMBs were mislocalized extracellularly.

Based on the RPKM values, two RNA sequences coded by LOC_Os09g00999 and LOC_Os09g01000 dominated the PMB transcriptome. Both aligned with the 18S and 26s rRNA sequences, suggesting that the ribosomes were a major constituent of the PMBs. The presence of ribosomes would be consistent with the transport and localization of RNAs as it is generally thought that mRNAs are transported in silenced polysome complexes. Alternatively, the presence of ribosomes may reflect autophagy, especially of rough ER-containing polysomes.

The composition of RNAs enriched on the PMBs was clearly non-random and was not simply a proportional sampling of the *glup6* transcriptome as RNAs that coded for specific classes of proteins were readily apparent. This was most apparent for the cell wall-related, respiration-energy, photosynthesis-energy, and ribosomal proteins classes, as well as for the miscellaneous protein class. The glycine-rich cell wall structural proteins comprised eight of the 10 RNAs in the cell wall-related class. Of the 38 RNAs in the miscellaneous class, 18 coded for ‘latency-associated nuclear antigen’ and six for a glycine-rich protein. RNA sequences of the two energy classes were also biased. In the respiration class, 13 RNAs coded for proteins involved in the F_1_ or F_0_ subunits of ATP synthase, two for cytochrome c oxidase, and two for NADH-ubiquinone oxido-reductase. Other than two RNAs coding for the Rubisco large subunit, all of the 31 remaining RNAs coded for subunits of PSI, PSII, or intermediates between these two photosystems (cytochrome b6 and plastocyanin), and for terminal NADPH-oxidoreductase.

The composition of the highly selective RNAs enriched on the PMBs (Supplementary Tables S2, S6) was not due to their specific expression levels in *glup6* compared to the wild-type. While there were RNAs that were not transcribed in wild-type developing seeds but activated in *glup6*, others showed relatively minor changes in gene expression compared with the wild-type (Supplementary Table S6). Likewise, the enrichment of these RNAs on the PMBs was not a product of chromosome location. While six of the glycine-rich cell wall-related proteins were clustered on chromosome 10, two other genes resided on chromosomes 6 and 11. Similarly, the locations of genes for ATP synthase were distributed on chromosomes 1, 4, 10, and 12. The presence of RNAs that coded for proteins involved in specific processes such as respiration or photosynthesis was consistent with the ‘regulon’ hypothesis (Keene, 2014) where sets of RNAs that share similar functions and/or intracellular locations are co-transported.

The activation of gene expression for proteins of the mitochondrial electron-transport chain inferred the need for energy production to support membrane trafficking. A greater requirement for energy was consistent with the increased membrane trafficking in *glup6* compared to the wild-type. The growth of the PMBs during seed development indicated that membrane vesicles were accumulated at this site and not recycled, necessitating the need for the biogenesis of new membrane, and hence ATP. In developing barley grains, oxygen gradients are readily apparent where oxygen levels are nearly depleted in the interior of the endosperm (Rolletschek *et al.*, 2004). Oxygen produced by photosynthesis in the outer green pericarp tissue of developing grains is utilized by respiration to release the energy required for storage-protein synthesis.

Similar to what was observed for glutelin, the RNAs for RBP-D, DEAD-box RNA helicase, DNA-dependent RNA polymerase subunit, and ubiquitin-conjugating enzyme were mislocalized to the PB-ER. Their mislocalization from the C-ER to the PB-ER in *glup6* suggested that they were transported by processes that shared many factors of the glutelin RNA transport pathway. Although all were secreted to the PMBs, the specificity of transfer as assessed by the RPKM-based PMB/*glup6* ratio varied from a high of 33-fold for RBP-D to a low of 0.04-fold for DEAD-box ATP-dependent RNA helicase, suggesting that these RNAs were transported by distinct pathways that were dependent on Rab5 and GEF. Likewise, the absence of an RNA encoding GTPase-activating protein on the PB-ER in *glup6* suggested that it used a different transport pathway to that taken by the others, e.g. a constitutive pathway.

The mislocalization of storage-protein RNAs mediated by defective Rab5a (Doroshenk *et al.*, 2010), Rab5a-GEF, and Got1B (Fukuda *et al.*, 2016) and the transport of mRNAs to the extracellular PMBs is consistent with RNA localization co-opting membrane trafficking events. While a close association between these processes has been suggested in metazoans (Gerst, 2008), substantial evidence for membrane-mediated RNA transport has been obtained in fungi and budding yeast cells (Schmid *et al.*, 2006; Kraut-Cohen and Gerst, 2010; Hermesh and Jansen, 2013; Haag *et al.*, 2015; Niessing *et al.*, 2018). In budding yeast, a subset of RNAs is co-transported with ER tubule membranes to the cortical region of the incipient daughter cells during the early phase of the cell cycle (Aronov *et al.*, 2007; Fundakowski *et al.*, 2012). A major factor in this ER-membrane association of mRNAs is She2p, which has dual RNA and ER membrane-binding properties. A second mechanism of co-transport of mRNAs with ER tubules occurs during pheromone signaling (Gelin-Licht *et al.*, 2012). Instead of She2p, this process requires Scp160p, a 140-kDa protein with multi-K homology RNA-binding domains (Frey *et al.*, 2001). In *Ustilago maydis*, RNAs are distributed throughout the growing hyphae on Rab5-associated early endosomes and are transported on microtubules (König *et al.*, 2009; Baumann *et al.*, 2012). Many of the players involved in this microtubule-based transport system have been identified including the endosome protein Upa1, which interacts with two RNA-binding proteins, Rrm4 and Pab1 (Pohlmann *et al.*, 2015).

An alternative mechanism for the delivery of mRNAs to the PMBs is by autophagy, especially ER-phagy. The loss of Rab5a activity would essentially prevent outward transport of membrane recycling from the plasma membrane, producing a significant imbalance in endomembranes and membrane-trafficking factors. In response to this stress, ER-phagy may be induced where subdomains of the C-ER form ER-phagosomes. Since the PMBs contain resident proteins of the ER, Golgi, prevacuolar compartment, and PSVs, they also probably contain lytic vacuole proteins as well. Instead of being delivered to the lytic vacuole, the ER-phagosome may be engulfed by the PMBs. The autophagic delivery of selective subdomains of the C-ER to the PMBs could account for the biased accumulation of sets of mRNAs in the PMBs if selective ER subdomains are prone to autophagy.

The increase in transcriptional activity in the *glup6* mutants compared to the wild-type (Table 1) was consistent with the significant changes that occurred at the cellular level. Elevated transcription of ABA/stress-related proteins, transcriptional factors, membrane proteins (mainly transporters), enzymes, and signal transduction components suggested that despite the significant disruption in membrane trafficking, the developing seed attempted to maintain homeostasis by activating transcription.

One apparent anomaly was the distribution of prolamine RNAs based on RNA-seq data of PMBs (Supplementary Table S7, storage proteins). Ten prolamine RNAs had RPKM values ≥5.0, ranging from 6.0 for LOC_Os06g31070.1 to >73 for LOC_Os07g10580.1. Moreover, α-globulin RNA was also found in RNA isolated from PMBs. As *in situ*-RT-PCR analysis clearly showed that the major prolamine and α-globulin RNAs were distributed to their usual location on the PB-ER in *glup6* (as well as *glup4*) and were not present on the PMBs, the presence of these storage-protein RNAs was probably an artifact of PMB purification. One plausible explanation is that the partially intact prolamine PBs, which contained peripherally bound prolamine and α-globulin RNAs, become associated with PMBs during their purification due to the hydrophobic nature of the exposed prolamine polypeptides. Whatever the explanation, our microscopic studies of rice endosperm sections (Fig. 4) showed that prolamine and α-globulin RNAs were only found on the PB-ER, their normal location, in *glup6* as well as *glup4*.

In view of their close biochemical relationship, loss of Rab5 and GEF activities would be expected to result in similar, if not identical changes in gene expression. In order to assess the degree of overlap between differentially regulated transcripts in developing seeds of *glup6* and *glup4*, the relevant subset of RPKM values from the *glup6* transcriptome analysis were compared with the fold-changes observed for differentially expressed genes identified in a previous microarray-based analysis of *glup4* developing seeds (Doroshenk *et al.*, 2010). In the *glup4* study, a total of 34 genes exhibited a 2-fold or greater increase in expression compared to the wild-type. Of these, 27 genes were also up-regulated (≥2.0) in *glup6* seeds based on their RPKM values (Supplementary Table S8). In contrast, there was essentially no correlation between the nine down-regulated genes identified in the microarray data for *glup4* and the RPKM values of the corresponding genes from the *glup6* RNA-seq analysis. Three of the down-regulated genes, LOC_Os09g39610, LOC_Os09g21500, and LOC_Os04g13364 (coding for expressed proteins) and LOC_Os12g39890 (coding for a transposon) were expressed in both *glup6* and the wild-type at RPKM values of ≤0.1.

There are a number of possible reasons as to why there was not a greater degree of overlap between the up-regulated genes in the *glup4* and *glup6* mutants. One factor likely to be important is that microarrays typically are not able to provide an accurate measure of fold-changes, especially for low-expression genes. This limitation comes from differences in hybridization efficiency as well as differences in cross-hybridization backgrounds among millions of array probes, while RNA-seq technology produces discrete, digital sequencing read counts and can quantify expression across a larger dynamic range (>10^5^ for RNA-seq versus 10^3^ for arrays) (Wang *et al.*, 2009; Wilhelm and Landry, 2009; Zhao *et al.*, 2014). It was not surprising, therefore, that the greater dynamic range of the RNA-seq method allowed a larger number of differentially regulated genes to be identified. Indeed, the greater dynamic range of RNA-seq showed that close to 4000 genes were up-regulated in *glup6* (Table 1) as assessed by RNA-seq compared with the 34 up-regulated genes in *glup4* identified by microarray analysis. It is likely that many more up-regulated genes would be identified in *glup4* using the greater sensitivity and dynamic range of RNA-seq.

A second, and likely compounding factor, is that rice contains a total of four Rab5 genes, two of which are conventional Rab5s (GLUP4/Rab5a and Rab5c) and two which are plant-specific (Rab5b and Rab5d), which are expressed during rice seed development. In addition, rice seeds also express two GEFs, GLUP6/GEF and Rab5-GEF2, (Wen *et al.*, 2015). It is likely that, collectively, these three additional Rab5s are better able to compensate for the lack of Rab5a (the major active isoform during seed development) in *glup4* than Rab5-GEF2 can compensate for the lack of GEF in *glup6*, thus explaining the greater number of transcriptional changes observed in the *glup6* mutant. Moreover, this could also be the basis for the apparent silencing of some genes (LOC_Os07g17310, B12D protein; LOC_Os03g19600, retrotransposons; and LOC_Os08g03720, transposon) that were up-regulated in *glup4* but silenced in *glup6*.

The specific roles of Rab5 and Rab5-GEF in the transport and localization of RNAs, including those for glutelin, remains to be resolved. Results from ongoing studies indicate that Rab5 interacts indirectly with RBP-P, the RNA-binding protein that recognizes and interacts with the glutelin zipcode sequences. Our current studies are now directed at resolving the nature of the multi-protein Rab5-RBP-P complex and its role in RNA transport and localization to the cisternal-ER.

## Supplementary data

Supplementary data are available at *JXB* online.

Table S1. Total transcriptome RPKM data for the wild-type), *glup6*, and for PMBs.

Table S2. Transcriptome RPKM data for genes that were up-regulated in *glup6* compared to the wild-type.

Table S3. Transcriptome RPKM data for genes that were down-regulated in *glup6* compared to the wild-type.

Table S4. Alignment of LOC_Os09g00999 and LOC_Os09g01000 cDNA sequences to various plant rRNA sequences.

Table S5. List of expressed proteins, hypothetical proteins, retrotransposons, and transposons enriched in PMBs.

Table S6. RNA classes enriched in PMBs with RPKM ratio ≥5.0.

Table S7. RNAs present in purified PMBs at RPKM ≥5.0.

Table S8. Comparison of up-regulated and down-regulated genes in *glup4* and *glup6*.

Table S9. List of primers used for *in situ* RT-PCR.

Fig. S1. Preparation of aleurone/subaleurone peels from developing rice seeds.

Fig. S2. Localization of selected RNAs on prolamine PBs and PMBs as assessed by *in situ* RT-PCR.

Supplementary Table S1

Supplementary Table S2

Supplementary Table S3

Supplementary Table S4

Supplementary Table S5

Supplementary Table S6

Supplementary Tables S7-S8 and Figures S1-S2

## Data deposition

Raw transcriptome data for the three biological repeats each for *glup6* and TC65 (wild-type) rice lines and the two biological repeats each for purified PMBs and seed peels are available at Dryad Digital Repository. doi:10.5061/dryad.sn878g4 (Yang *et al.*, 2018).
