# Supplementary material for: Selective sets of mRNAs localize to extracellular paramural bodies in a rice glup6 mutant
Source: J Exp Bot. 2018 Aug 9;69(21):5045–58. doi: 10.1093/jxb/ery297 (PMC6189835; doi:10.1093/jxb/ery297)
Supplement: Supplementary Tables S7-S8 and Figures S1-S2 [file ery297_suppl_supplementary_tables_s7-s8_figures_s1-s2.pdf]

Supplementary Table S7 - Comparison of up-regulated and down-regulated genes in *glup4* and *glup6.*

Genes highlighted in red differ significantly in expression between glup4 and glup6. #DIV/0! denotes that the gene is in glup6 but is silenced in WT.

gene ID	*glup4* /WT microarray	*glup6* /WT RNA-seq	WT avgRPKM	*glup6* avgRPKM	Putative model
LOC_Os05g39600	6.6	21.3	0.07	1.45	ATCHX15,
LOC_Os01g72900	5.3	99.2	0.52	51.99	abscisic stress-ripening,
LOC_Os08g38710	4.7	546.0	0.05	0.78	uncharacterized glycosyltransferase,
LOC_Os09g39960	3.9	#DIV/0!	0.00	1.52	dynamin family protein,
LOC_Os11g37950	3.5	#DIV/0!	0.00	5.19	WIP3 - Wound-induced protein precursor,
LOC_Os09g14610	3.5	17.1	0.13	2.18	DCL2,
LOC_Os12g29400	3.3	17.6	0.36	6.36	GRAM domain containing protein,
LOC_Os07g17310	3	0.0	0.00	0.00	B12D protein,
LOC_Os07g17300	2.9	20.4	0.10	2.12	protein
LOC_Os06g13190	2.9	71.2	0.25	17.81	protein
LOC_Os01g71080	2.7	5.3	0.73	3.87	xylanase inhibitor,
LOC_Os01g34410	2.6	5.4	2.27	12.34	transposon protein, putative, Pong sub-class,
LOC_Os07g39970	2.5	7.6	5.41	40.84	ZOS7-08 - C2H2 zinc finger protein,
LOC_Os03g19600	2.5	0.0	0.03	0.00	retrotransposon protein, putative, unclassified
LOC_Os08g42890	2.5	1.5	0.55	0.82	pectinesterase inhibitor domain containing protein
LOC_Os04g54230	2.5	1.4	37.18	50.95	wound induced protein
LOC_Os02g56810	2.4	#DIV/0!	0.00	1.25	OsFBX68 - F-box domain containing protein
LOC_Os06g32730	2.4	#DIV/0!	0.00	0.13	eukaryotic initiation factor iso-4F subunit p82-34
LOC_Os08g04130	2.4	15.4	3.83	58.89	copine-6
LOC_Os10g35460	2.3	19.9	0.37	7.30	COBRA
LOC_Os01g62460	2.3	12.1	1.05	12.69	ZOS1-16 - C2H2 zinc finger protein
LOC_Os08g03720	2.3	0.0	0.01	0.00	transposon protein, putative, CACTA, En/Spm sub-class
LOC_Os05g09500	2.3	4.6	0.36	1.65	hexokinase,
LOC_Os09g03190	2.2	1.3	1.52	1.98	protein
LOC_Os08g01610	2.2	4.8	0.30	1.41	DUF250 domain containing protein
LOC_Os10g38820	2.2	18.4	0.31	5.62	bZIP family transcription factor
LOC_Os02g31030	2.2	1.0	25.92	26.14	glycerophosphoryl diester phosphodiesterase family protein
LOC_Os05g51390	2.1	3.8	0.51	1.93	uncharacterized protein PA4923
LOC_Os10g40640	2	7.8	0.11	0.86	glycosyl transferase 8 domain containing protein
LOC_Os02g46650	2	3.2	0.31	1.00	ubiquitin carboxyl-terminal hydrolase domain
LOC_Os10g37760	2	7.9	0.98	7.70	OsRhmbd17 - Putative Rhomboid homologue
LOC_Os03g60560	2	12.5	0.81	10.13	ZOS3-21 - C2H2 zinc finger protein
LOC_Os06g51320	2	4.8	0.55	2.65	Gibberellin-regulated GASA/GAST/Snakin family protein
LOC_Os03g55070	2	8.3	7.68	63.89	UDP-glucose 6-dehydrogenase
LOC_Os03g48810	−3.1	1.2	11.43	13.41	nucleobase-ascorbate transporter,
LOC_Os04g49890	−2.8	1.7	1.76	2.96	multidrug resistance-associated protein
LOC_Os09g39610	−2.7	0.0	0.00	0.10	expressed protein
LOC_Os09g21500	−2.6	0.0	0.00	0.00	expressed protein
LOC_Os04g13364	−2.3	1.3	0.08	0.10	transposon protein, CACTA, En/Spm sub-class
LOC_Os09g19820	−2.3	1.9	0.32	0.62	aminopeptidase,
LOC_Os11g09460	−2.2	1.3	0.94	1.19	expressed protein
LOC_Os11g08590	−2.0	2.9	0.17	0.49	expressed protein
LOC_Os12g39890	−2.0	1.0	0.08	0.08	expressed protein

Supplementary Table S8. Primer list used in *in situ* RT-PCR

Rice locus	Gene Product Name	Primer (5'→3')	Note
LOC_Os01g55690	Glutelin	ccctcaagcatacaggcgtg	F
		cgctctcttgattgcacttgtcc	R
LOC_Os07g10570	Prolamine	aatgcctctgcgcggtttgatcc	F
		caccaacagtggcaatgctc	R
LOC_Os05g41790	α-globulin	ggagaggttccagccgatgtt	F
		cgtagtacccctgctccgac	R
LOC_Os06g37000	RNA binding protein D	gcaattctggaagcagca	F
		cggtgcaccatatgcaccacc	R
LOC_Os03g57790	Ubiquitin-conjugating enzyme	tgatgcgggacttcaagcg	F
		ttccagagcatgatgttgttgtc	R
LOC_Os01g59850	GTPase-activating protein	ggagaggaacgagaagatca	F
		tggtacatacatattgtggcc	R
LOC_Os06g48750	DEAD-box ATP-dependent RNA helicase	cgggaatggcaccagag	F
		gatgtgaagaactcctcggt	R
LOC_Os04g16820	DNA-directed RNA Polymerase subunit β	aattatacgtatccgcgcgattaatt	F
		caatatttgattgatcacaattcgg	R

The “aleurone-subaleurone peel” method

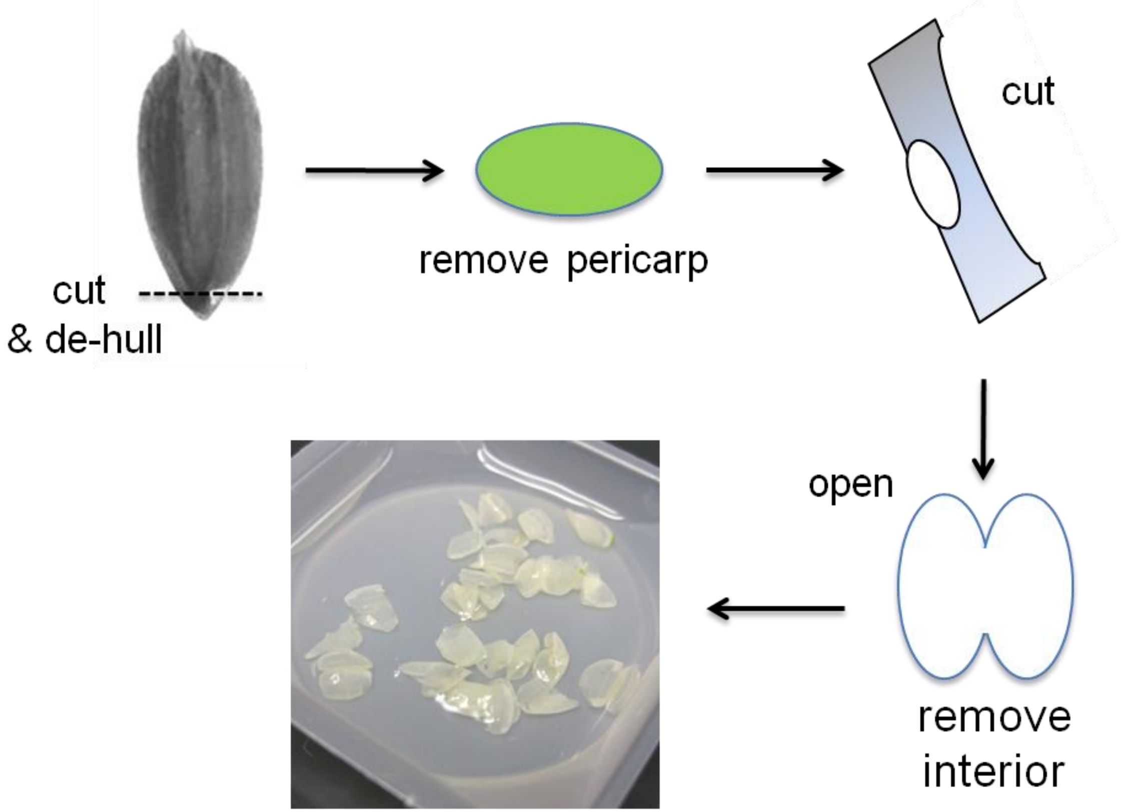

Supplementary Fig. S1. Preparation of the aleurone-subaleurone peel method.
In order to prepare “aleurone-subaleurone peels”, the pericarp was removed from mid-developing rice seeds using tweezers and seeds were sliced longitudinally using a thin razor blade leaving 1 mm of tissue uncut (so that the seeds resembled a book with the uncut portion being the spine). The two halves of the seed were then opened up and the soft bulky endosperm tissue removed to leave the tegmentum, the aleurone cell layer and 3-5 layers of subaleurone cells intact and ready for fluorescent labeling.

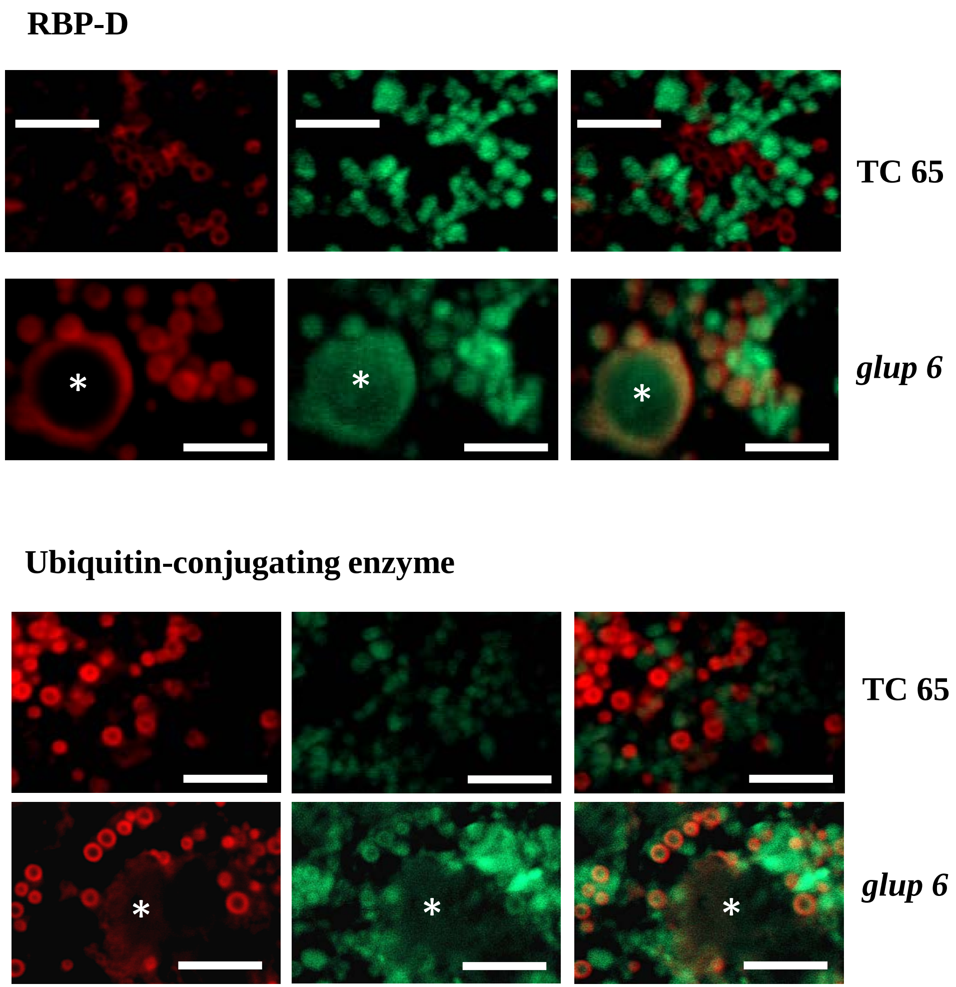

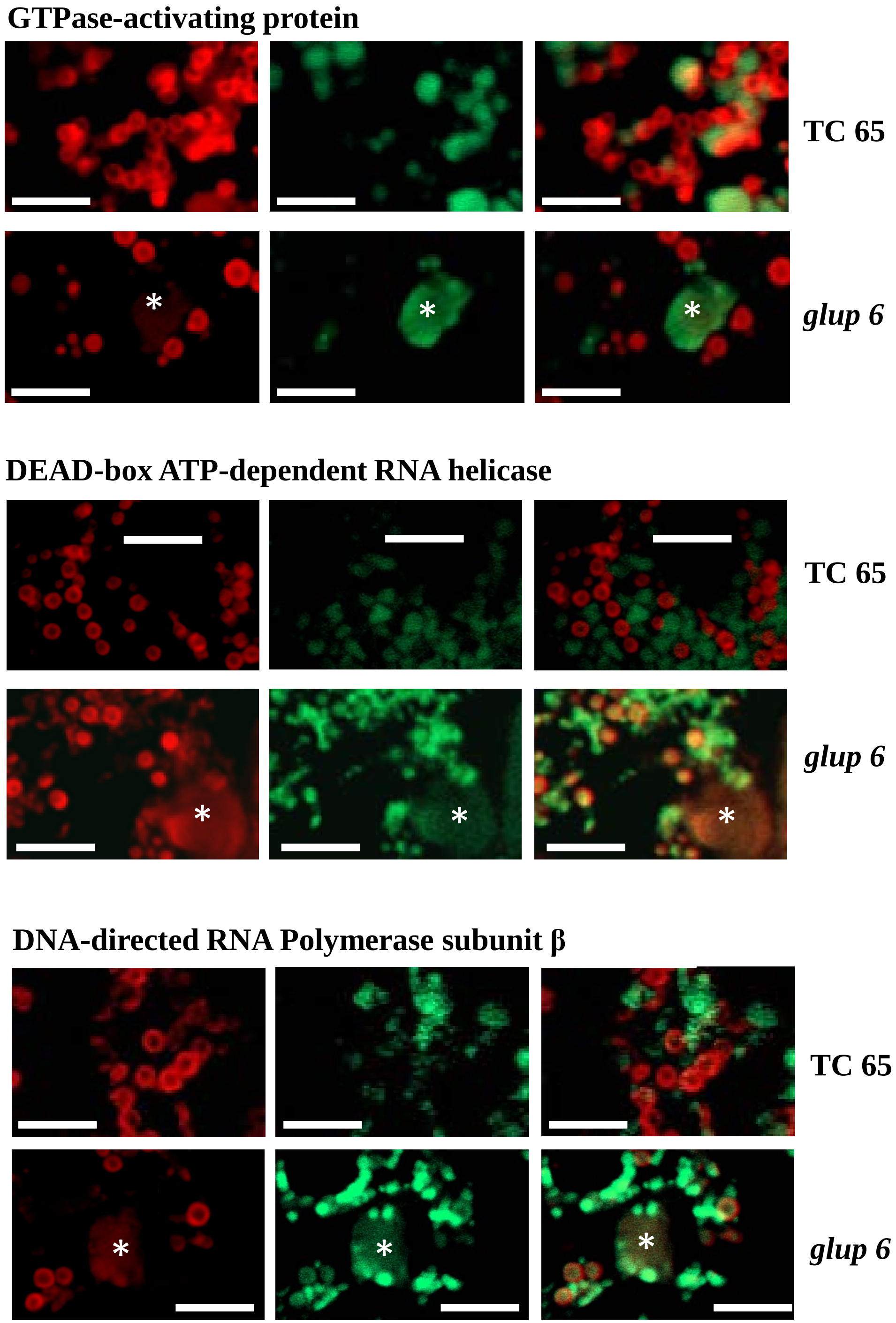

Supplementary Fig. S2. Mis-localization of various RNAs to prolamine PBs and PMBs in *glup6* compared to their normal location on the cisternal-ER in wildtype TC65. The left panel of each series depicts the staining of prolamine PBs and PMBs by Rhodamine (red), the middle panel shows the distribution of glutelin RNAs (green) as assessed by *in situ* RT-PCR, while the right panel represents the merged images of the left and middle panel in each series. The PMBs in each *glup6* panel are labeled with an *. Bar = 5 µm.
